# Peripheral Effects of the Gut-Derived Metabolite TMAO in Two MPTP-Challenged Disease Animal Models

**DOI:** 10.3390/ijms27209377

**Published:** 2026-10-21

**Authors:** Paul-Ștefan Panaitescu, Vlad-Alexandru Toma, Claudia-Andreea Moldoveanu, Remus Moldovan, Ioana Bâldea, Irina Camelia Haranguș, Carmen Costache, Simona Clichici, Gabriela-Adriana Filip

**Affiliations:** 1 Department of Microbiology, “Iuliu Hatieganu” University of Medicine and Pharmacy, 400349 Cluj-Napoca, Romania; 2 Department of Medical and Health Sciences, Babes-Bolyai University, 400394 Cluj-Napoca, Romania; 3 Institute of Biological Research Cluj, National Institute of Research and Development for Biological Sciences, 400015 Cluj-Napoca, Romania; 4 Department of Molecular Biology and Biotechnology, Babes-Bolyai University, 400008 Cluj-Napoca, Romania; 5 Department of Physiology, “Iuliu Hatieganu” University of Medicine and Pharmacy, 400006 Cluj-Napoca, Romaniaichis@umfcluj.ro (I.C.H.); sclichici@umfcluj.ro (S.C.); 6 Department of Anatomy and Embriology, “Iuliu Hatieganu” University of Medicine and Pharmacy, 8 Victor Babeş Street, 400012 Cluj-Napoca, Romania; gabriela.filip@umfcluj.ro

**Keywords:** TMAO, MPTP, gut–liver–brain axis, kidney–brain axis, inflammation, oxidative stress

## Abstract

Parkinson’s disease (PD) remains incompletely understood despite its high social and economic burden, with oxidative stress, neuroinflammation, and mitochondrial dysfunction recognized as central pathogenic mechanisms. 1-methyl-4-phenyl-1,2,3,6-tetrahydropyridine (MPTP), a complex I inhibitor, is widely used as a model of dopaminergic neurodegeneration, while growing evidence highlights the gut–liver–brain and kidney–brain axes as important contributors to PD progression. Within this framework, trimethylamine N-oxide (TMAO) has emerged as a molecule of interest, linked to dementia risk and proinflammatory effects. Our study aimed to evaluate the peripheral oxidative and inflammatory consequences of acute and semi-acute MPTP exposure, alone and in combination with TMAO. In total, 96 male CD-1 mice were equally divided across two experimental settings: a semi-acute protocol (30 mg/kg i.p., MPTP once daily for five consecutive days) and an acute protocol (20 mg/kg i.p., four administrations of MPTP at two-hour intervals), with TMAO administration remaining identical across both experimental settings. The animals were randomized into four groups (control group, a TMAO-only group (40 mg/kg/day, gavage, days 0–42), an MPTP-only group, and a combined TMAO + MPTP group), with MPTP showing consistently depleted liver SOD activity across both models. In the liver, MPTP showed consistently depleted SOD activity in both models, whereas glutathione disruption and lipid peroxidation were most evident in the combined TMAO + MPTP group. These effects were explained mainly by the additive main effects of MPTP and TMAO, as no significant MPTP × TMAO interaction was found for any oxidative stress marker. Nonetheless, a significant MPTP × TMAO interaction was confirmed for liver and kidney IL-1β in the acute model. IL-1β and IL-6 were significantly elevated in the liver and kidney by both MPTP alone and its combination with TMAO, with the combined groups consistently showing the largest increases (TMAO + MPTP semi-acute vs. control and vs. TMAO, *p* < 0.001–0.01; TMAO + MPTP acute vs. all groups, *p* < 0.0001–0.01). In the kidney, the contribution of MPTP and TMAO to oxidative stress was model-dependent: in the semi-acute protocol, both MPTP and TMAO contributed to increased MDA and glutathione ratio disruption, while in the acute protocol, TMAO alone was the dominant driver of SOD depletion and MDA elevation, with their combination further amplifying IL-6 secretion. TMAO also independently drove lipid peroxidation in the heart and intestine. The acute MPTP model produced more extensive redox imbalance independently of TMAO, whereas in the semi-acute model, a comparable degree of redox disruption was only achieved through combined TMAO + MPTP exposure.

## 1. Introduction

PD poses a high economic and social burden [1]; however, the full pathogenicity of PD is yet to be completely elucidated. Given this need for exploration, animal models are frequently used to identify the molecular interactions and mechanistic pathways that could explain different steps in the development and progression of the disease. As of now, the known contributors to the pathogenic process are an inflammatory response coupled with oxidative damage [2,3], with dopaminergic lesions developing due to oxidative imbalances, neuroinflammation, and energetic metabolism impairment [4,5]. The aforementioned ROS can be exogenous [6] or have an endogenous source based on respiratory chain alteration in the mitochondria [7]. A reduction in complex I of the respiratory chain [8] has been proven to increase ROS, a fact validated by the use of complex I inhibitors such as MPTP for inducing cytotoxicity in dopaminergic neurons and PD-like symptoms [9]. Gut health also plays an increasingly important role in the development of the disease [5,10]. More recent models of the disease are expanding on how α-syn is transported from the gut to the brain and the other way around [11,12]. Certain gut metabolites have risen to prominence as possible important contributors to PD’s progression—one such metabolite is trimethylamine (TMA) and its hepatically oxidized form, trimethylamine N-oxide (TMAO) [13].

Choline, betaine, L-carnitine, and γ-butyrobetaine can all be metabolized at the gut microbiota level into TMA, which is then absorbed systemically [14]. TMA is subsequently metabolized at the hepatic level into TMAO through flavin monooxygenase (FMO) 1 and 3 [15], after which TMAO is predominantly excreted in urine [16]. This dual dependence on hepatic biotransformation and renal clearance places the liver and kidney at the center of TMAO’s systemic handling and, by extension, at the center of its potential downstream effects on PD pathogenesis. Given the important role that the liver plays in the metabolism of gut-derived molecules, it is considered that the gut–brain axis would be better described as the the gut–liver–brain axis [17].

This liver- and kidney-dependent handling of TMAO is not merely a pharmacokinetic detail—both organs are increasingly implicated as active contributors to, rather than passive bystanders of, PD progression. A population-based study found that TMAO increases the risk of dementia, with precursors of TMAO being associated with poor cognition [18]. Because renal clearance is central to the elimination of this osmolyte, the same study assessed how kidney function affects TMAO concentrations and their association with dementia risk, finding that impaired renal clearance leads to increased dementia risk with elevated TMAO, while in those with preserved renal function, only TMAO precursor levels were related to dementia risk [18]. These findings are of high relevance, as kidney function is increasingly proposed as an additional piece of the Parkinson’s pathogenesis puzzle given its role in the clearance of α-syn [19] and proinflammatory molecules such as TMAO [20] and its involvement in glucose metabolism impairment [21,22]. Recent research has anatomically and functionally confirmed parasympathetic innervation of the kidney, contrary to the earlier literature [23], and has suggested that, analogous to α-syn’s vagal transit from the intestine to the brain, a comparable kidney-to-brain route may exist, raising the possibility of a kidney–brain axis alongside the well-established gut–brain axis [24,25]. TMAO is also a well-known pro-atherosclerotic molecule and is implicated in heart failure through multiple mechanisms [26], further extending its relevance beyond the liver–kidney axis to other peripheral organs.

Despite this expanding evidence base implicating peripheral organs—liver, kidney, and beyond—in TMAO handling and PD-relevant pathology, a critical gap remains: the peripheral effects of MPTP itself remain largely uncharacterized. Importantly, in vivo MPTP oxidation by FMOs is still debated, with an older study finding N-oxidation by rat liver FMOs [27] and a marmoset in vivo study showing the role of FMO3 in the deactivation of dopaminergic toxic metabolites of MPTP [28]. MPTP models are frequently used to induce experimental PD, with multiple regimens—acute, semi-acute, and chronic. Our study focuses on the two most frequently used regimens, acute (20 mg/kg bw, i.p., four times at 2-h intervals) and semi-acute (30 mg/kg bw, i.p., once daily, for 5 consecutive days) [29].

The rationale for examining peripheral oxidative stress and inflammation across the liver, kidney, heart, and intestine is grounded in several converging mechanisms of direct relevance to PD progression. Systemically elevated proinflammatory cytokines, particularly IL-1β and IL-6, can compromise blood–brain barrier integrity and activate central neuroinflammatory cascades [30]. The oxidative imbalances under investigation—encompassing lipid peroxidation, glutathione depletion, and antioxidant enzyme disruption—engage the NF-κB and Nrf2-Keap1 signaling axes, as well as the NLRP3 inflammasome [31,32], both of which are central regulators of peripheral and central inflammatory responses in PD. Furthermore, oxidative stress itself promotes α-syn misfolding and aggregation, establishing a self-amplifying cycle between peripheral redox imbalance and central neurodegeneration [32]. Intestinal oxidative stress, in particular, is positioned at the interface of the gut–brain axis, where inflammatory signals and pathological α-syn can propagate toward the CNS via vagal and systemic routes [33]. Collectively, these findings suggest that peripheral organ involvement in PD is not merely a secondary consequence of central neurodegeneration but may represent an active and bidirectional contributor to disease progression.

Despite this expanding evidence on the relationships and axes linking peripheral organs to the brain, no study to date has systematically evaluated the combined effects of TMAO and MPTP on oxidative stress and proinflammatory cytokines across multiple peripheral organs simultaneously. This represents a significant gap given that TMAO and MPTP share a common hepatic oxidation pathway and exert overlapping downstream cellular effects, yet their potential interaction at the level of peripheral organ redox and inflammatory status remains entirely unexplored. Addressing this gap, the present study provides, to our knowledge, the first systematic assessment of how an atherosclerotic dose of TMAO modulates MPTP-induced oxidative stress and inflammatory responses in the liver, kidney, heart, and intestine across both acute and semi-acute MPTP regimens. Specifically, we aimed to determine (i) whether TMAO alone induces peripheral oxidative and inflammatory changes independent of MPTP exposure, (ii) whether MPTP alone affects these same peripheral organs, and (iii) whether the combination of TMAO and MPTP produces synergistic effects beyond those observed with either compound alone, which would support a link between gut-derived metabolite handling, peripheral organ dysfunction, and the pathophysiology of PD.

## 2. Results

### 2.1. Oxidative Stress Markers

#### 2.1.1. Liver

**Figure 1:**
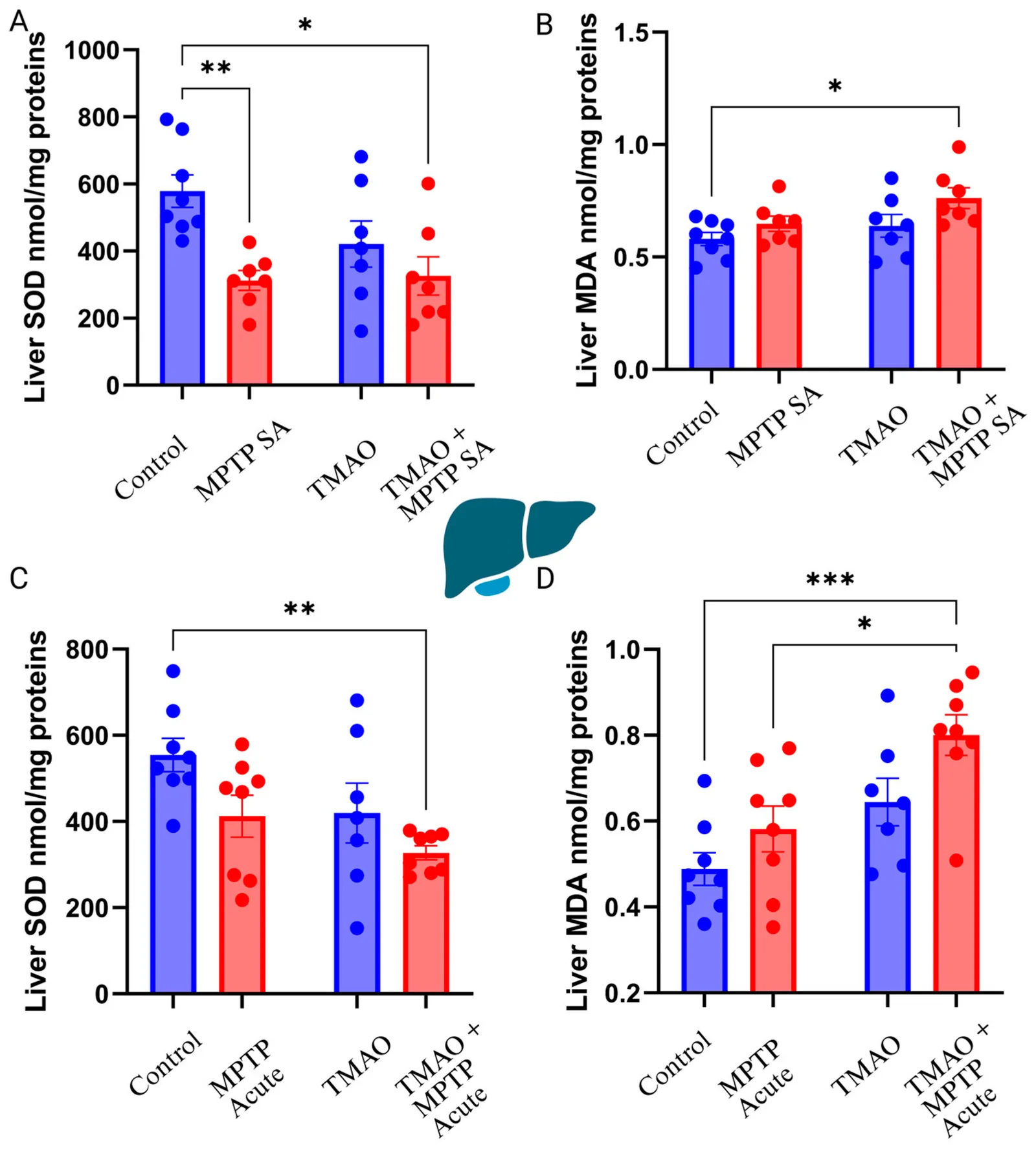
Liver SOD activity and MDA levels in MPTP semi-acute model (**A**,**B**) (control *n* = 8; MPTP SA *n* = 7; TMAO *n* = 7; TMAO + MPTP SA *n* = 7) and acute model (**C**,**D**), (control *n* = 8; MPTP acute *n* = 8; TMAO *n* = 7; TMAO + MPTP acute *n* = 8), two-way ANOVA and Tukey’s honestly significant difference (HSD) post hoc test. For the first experiment, SOD activity decreased significantly in the liver compared to controls for MPTP SA (*p* < 0.01), TMAO + MPTP SA (*p* < 0.05) (**A**). Liver MDA levels were increased in TMAO + MPTP SA (*p* < 0.05) compared to controls (**B**). In the acute model, SOD was lowered vs. control by TMAO + MPTP acute (*p* < 0.01). (**C**) MDA level increase was noticed in acute experiment when TMAO + MPTP was compared with control (*p* < 0.001) and MPTP acute (*p* < 0.05) (**D**). Results are expressed as mean ± SEM, * *p* < 0.05, ** *p* < 0.01, *** *p* < 0.001.

**Figure 2:**
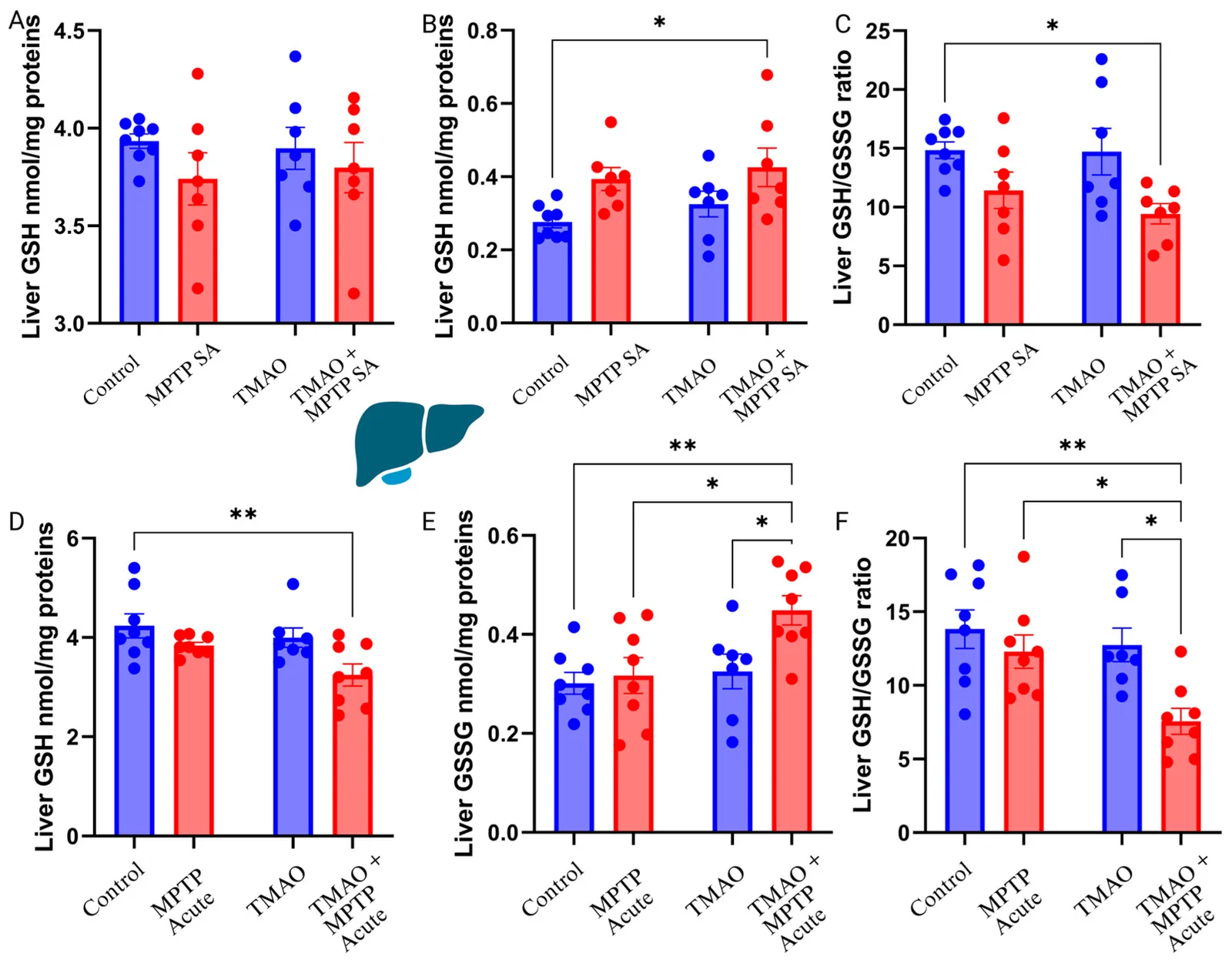
Liver gluthatione homeostasis. Liver levels of GSH and GSSG and GSH/GSSG ratio in MPTP SA (**A**–**C**) (control *n* = 8; MPTP SA *n* = 7; TMAO *n* = 7; TMAO + MPTP SA *n* = 7) and MPTP acute model (**D**–**F**) (control *n* = 8; MPTP acute *n* = 8; TMAO *n* = 7; TMAO + MPTP acute *n* = 8), two-way ANOVA and Tukey’s honestly significant difference (HSD) post hoc test. No statistically significant difference was found for liver GSH in the semi-acute model (**A**), with the combined administration leading to an increase in liver GSSG, TMAO + MPTP SA vs. control, *p* < 0.05, (**B**) and lowered GSH/GSSG ratio in the same group (TMAO + MPTP SA vs. control, *p* < 0.05) (**C**). The acute model revealed a significant (*p* < 0.01) reduction in GSH in TMAO + MPTP acute group compared to the control (*p* < 0.01) (**D**), with the same group increasing GSSG vs. control (*p* < 0.01) vs. MPTP acute (*p* < 0.05) and vs. TMAO (*p* < 0.05). (**E**). The ratio of GSH/GSSG was lowered in the TMAO + MPTP acute vs. control (*p* < 0.01), vs. MPTP acute (*p* < 0.05) and vs. TMAO (*p* < 0.05) (**F**). Results are expressed as mean ± SEM, * *p* < 0.05, ** *p* < 0.01.

The MPTP semi-acute (SA) disease model produced liver redox imbalances, partially depleting SOD activity (F(1, 25) = 11.78, *p* = 0.0021) (Figure 1A. MPTP SA vs. control, *p* < 0.01; TMAO + MPTP SA vs. control, *p* < 0.05). The same model (F(1, 25) = 5.575, *p* = 0.0263) increased MDA in the TMAO + MPTP SA group (Figure 1B, *p* < 0.05 vs. control), but it only accounted for part of the effect, as the intervention also explained part of the variation (F(1, 25) = 4500, *p* = 0.0440). No effect was observed in the case of GSH (Figure 2A), but the semi-acute model (F(1, 25) = 9617, *p* = 0.0047) led to an effect in the TMAO + MPTP SA group, increasing GSSG (Figure 2B, *p* < 0.05 vs. control). The inverse effect (F(1, 25) = 10.52, *p* = 0.0033) was observed in the lowered GSH/GSSG ratio (Figure 2C, *p* < 0.05 vs. control).

In the case of the MPTP acute model, it depleted SOD (F(1, 27) = 6611, *p* = 0.0160), with the intervention also doing the same (F(1, 27) = 5797, *p* = 0.0232), and increased MDA (F(1, 27) = 6545, *p* = 0.0164), again with the intervention accounting for part of the effect (F(1, 27) = 14.76, *p* = 0.0007) ((Figure 1C. MPTP acute vs. control, *p* < 0.05; TMAO + MPTP acute vs. control, *p* < 0.001) vs. MPTP acute (*p* < 0.05)) (Figure 1D). GSH was depleted partially due to the model (F(1, 27) = 8763, *p* = 0.0063) and partially due to the intervention (F(1, 27) = 4551, *p* = 0.0421) (Figure 2D, TMAO + MPTP acute vs. control, *p* < 0.01). Comparable results were observed for GSSG, with the model and the intervention increasing it (Figure 2E, TMAO + MPTP acute vs. control, *p* < 0.01 vs. MPTP acute, *p* < 0.05, vs. TMAO, *p* < 0.05). For the GSH/GSSG ratio, it was reduced by both the disease (F(1, 27) = 8792, *p* = 0.0063) and the intervention (F(1, 27) = 6567, *p* = 0.0163) (Figure 2F, TMAO + MPTP SA vs. control, *p* < 0.001, vs. MPTP SA, *p* < 0.05, vs. TMAO *p* < 0.05).

The TMAO intervention on its own did not produce effects on the liver oxidative stress; it did account, in part, for the variation in the case of SOD and MDA and for gluthatione homeostasis in the second experiment and MDA in the first experiment. The disease model and the intervention did not have any statistically significant interaction with liver oxidative stress.

#### 2.1.2. Kidney

**Figure 3:**
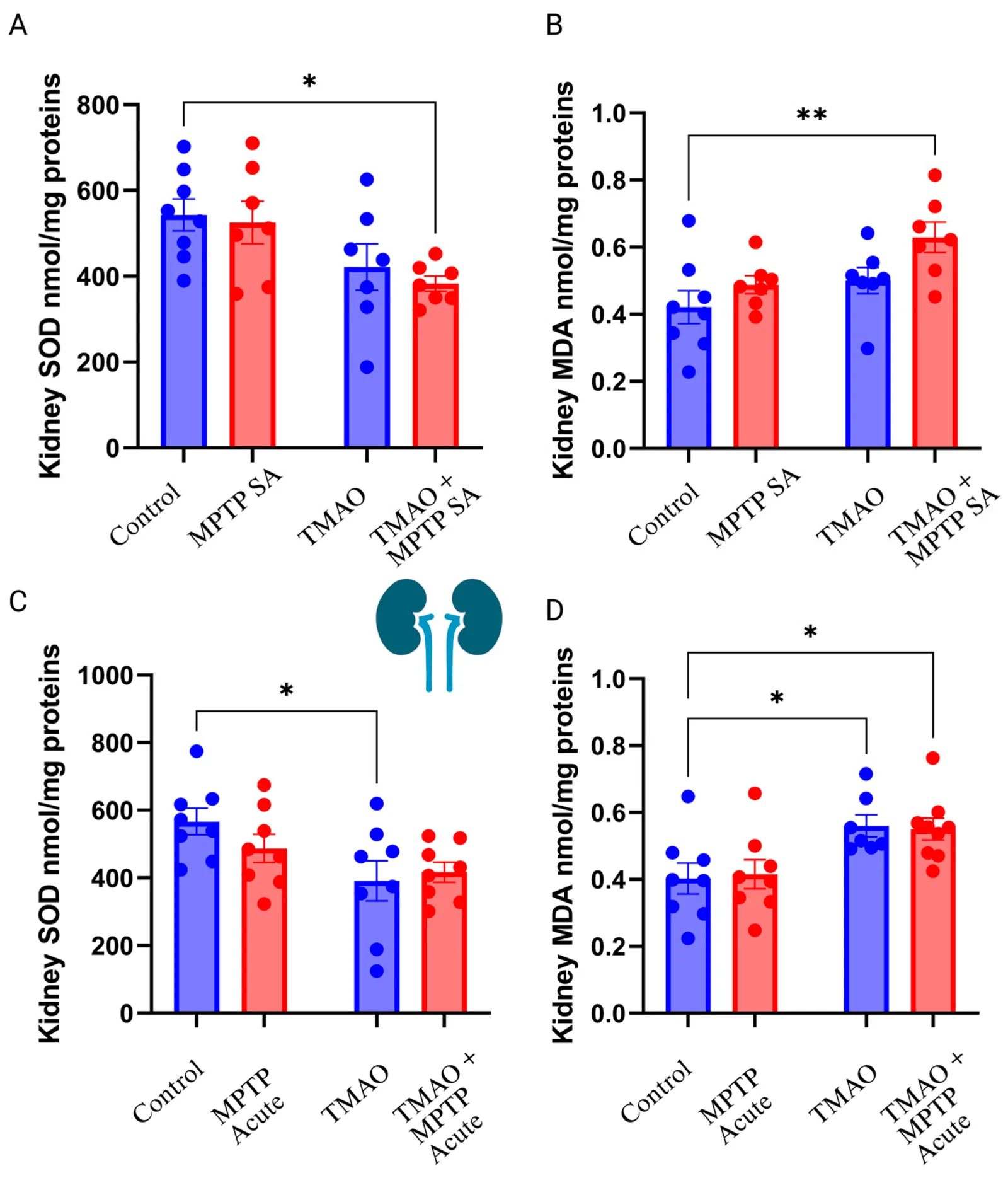
Kidney SOD activity and MDA levels in MPTP semi-acute model (control *n* = 8; MPTP SA *n* = 7; TMAO *n* = 7; TMAO + MPTP SA *n* = 7) (**A**,**B**) and acute model (control *n* = 8; MPTP acute *n* = 8; TMAO *n* = 7; TMAO + MPTP acute *n* = 8) (**C**,**D**), two-way ANOVA and Tukey’s honestly significant difference (HSD) post hoc test. In the semi-acute model, kidney SOD activity (**A**) was depleted by TMAO + MPTP SA compared to control (*p* < 0.05). Kidney MDA levels increased in TMAO + MPTP SA vs. control (*p* < 0.01) (**B**). For the acute model, SOD (**C**) activity decreased in TMAO group (*p* < 0.05). MDA increase was noticed in acute experiment and in TMAO (*p* < 0.05) and TMAO + MPTP acute (*p* < 0.05) compared to control (**D**). Results are expressed as mean ± SEM, * *p* < 0.05, ** *p* < 0.01.

**Figure 4:**
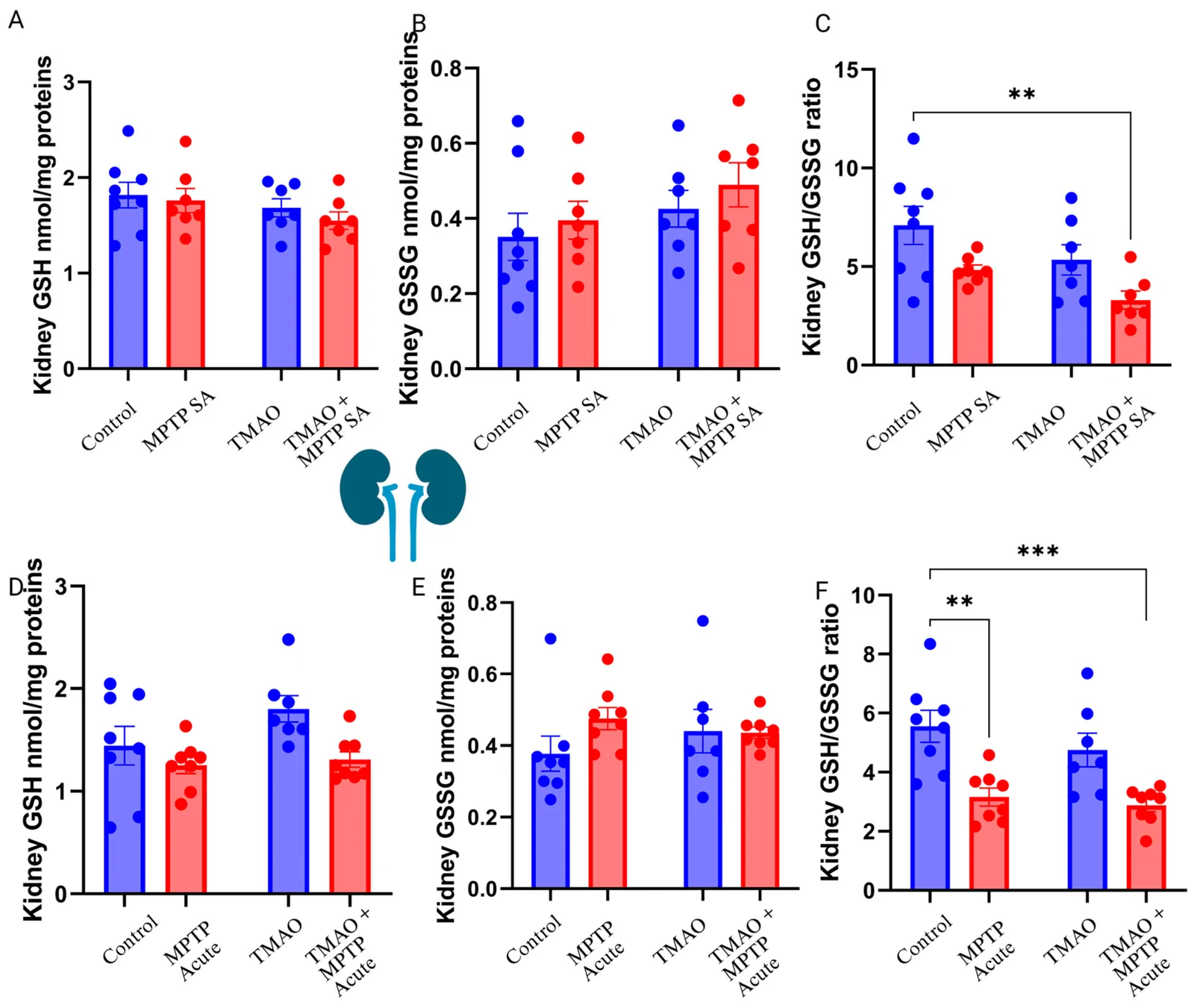
Kidney glutathione homeostasis. Kidney levels of GSH and GSSG and GSH/GSSG ratio in MPTP SA (control *n* = 8; MPTP SA *n* = 7; TMAO *n* = 7; TMAO + MPTP SA *n* = 7) (**A**–**C**) and MPTP acute model (control *n* = 8; MPTP acute *n* = 8; TMAO *n* = 7; TMAO + MPTP acute *n* = 8) (**D**–**F**), two-way ANOVA and Tukey’s honestly significant difference (HSD) post hoc test. No statistically significant alteration of GSH levels (**A**) and GSSG (**B**) was found in semi-acute model. Nonetheless, GSH/GSSG ratio was decreased in TMAO + MPTP SA group vs. control (*p* < 0.01) (**C**). No statistically significant reduction in GSH levels (**D**) and GSSG levels (**E**) was found in the acute model. TMAO + MPTP acute (*p* < 0.001) and MPTP acute (*p* < 0.01) vs. control led to decreases in the GSH/GSSG ratio (**F**). Results are expressed as mean ± SEM, ** *p* < 0.01, *** *p* < 0.001.

In the kidney, the TMAO intervention led to more oxidative stress than in the liver. In the case of the first experiment, it led to depleted SOD (F(1, 25) = 9968, *p* = 0.0041). SOD activity was depleted in groups that received TMAO compared to control (Figure 3A, *p* < 0.05). TMAO also increased MDA (F(1, 25) = 6947, *p* = 0.0142) but in addition to the disease model (F(1, 25) = 5509, *p* = 0.0271). MDA was increased in the same groups (Figure 3B, TMAO + MPTP SA vs. control, *p* < 0.01 vs. MPTP, *p* < 0.05). No effect was observed in the case of GSH (Figure 4A) and GSSG (Figure 4B). The same effects caused by the model (F(1, 25) = 9413, *p* = 0.0051) and the intervention (F(1, 25) = 5443, *p* = 0.028) resulted in lowered GSH/GSSG ratio (Figure 4C, *p* < 0.01 vs. control).

In the second experiment, similar results were observed, with TMAO being the sole cause of SOD (F(1, 27) = 7793, *p* = 0.0093) and MDA (F(1, 27) = 13.70, *p* = 0.0009) alterations. SOD activity was depleted in the TMAO group compared to control (Figure 3C, *p* < 0.05) while the TMAO + MPTP SA group was not significant (*p* = 0.0985). MDA was increased in both groups (Figure 3D, TMAO + MPTP acute vs. control, *p* < 0.05 vs. MPTP acute, *p* < 0.05). No effect was observed in the case of GSH (Figure 4D), but the acute model (F(1, 27) = 7243, *p* = 0.0121) led to an effect in the TMAO + MPTP SA group, with increasing GSSG (Figure 4E, TMAO + MPTP acute vs. control, *p* < 0.05). The same effects caused by the model (F(1, 27) = 25.07, *p* < 0.0001) resulted in lowered GSH/GSSG ratio (Figure 4E, TMAO + MPTP acute vs. control, *p* < 0.001, and vs. TMAO, *p* < 0.05, MPTP acute vs. control, *p* < 0.01).

The disease model did not have any statistically significant interaction with the intervention in the case of oxidative stress markers in the kidney.

#### 2.1.3. Heart and Intestine

**Figure 5:**
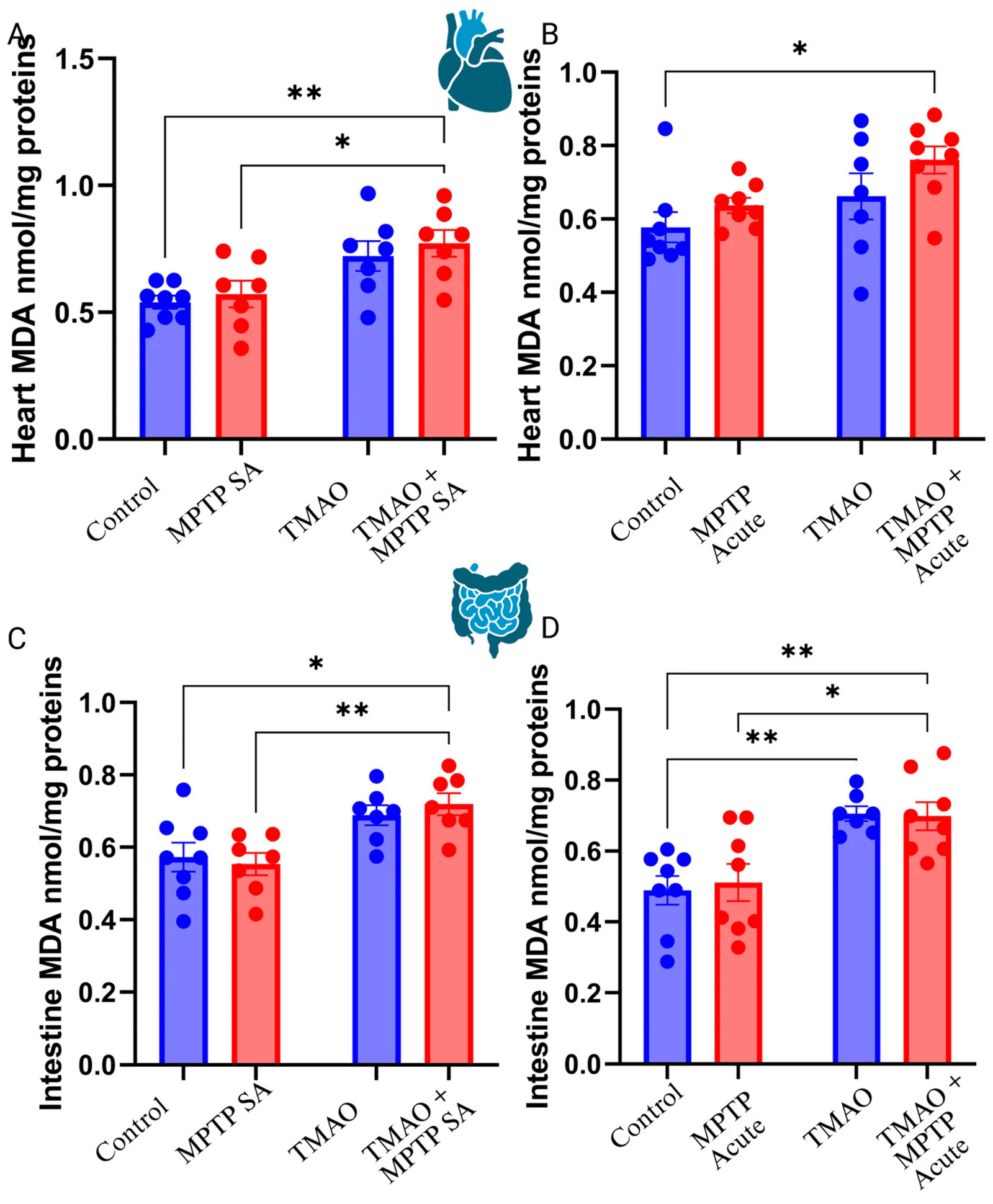
Heart and intestine MDA. Two-way ANOVA and Tukey’s honestly significant difference (HSD) post hoc test. In the case of heart tissue, in the semi-acute model (**A**) (control *n* = 8, MPTP SA *n* = 7, TMAO *n* = 7, TMAO + MPTP SA *n* = 7), TMAO + MPTP increased MDA vs. control (*p* < 0.001) and vs. MPTP SA (*p* < 0.05). In the acute model (**B**) (control *n* = 8, MPTP acute *n* = 8, TMAO *n* = 7, TMAO + MPTP acute *n* = 8), TMAO + MPTP acute increased heart MDA vs. control (*p* < 0.05). In the intestine, in semi-acute model (**C**) (control *n* = 8, MPTP SA *n* = 7, TMAO *n* = 7, TMAO + MPTP SA *n* = 7), the TMAO + MPTP SA group showed increased MDA vs. control (*p* < 0.05), as well as vs. MPTP acute (*p* < 0.01). In the acute model (**D**) (control *n* = 8, MPTP acute *n* = 8, TMAO *n* = 7, TMAO + MPTP acute *n* = 8), TMAO led to increased MDA vs. control, both in the TMAO group (*p* < 0.01) and the TMAO + MPTP acute group (*p* < 0.01). TMAO + MPTP acute also led to increased values vs. MPTP SA (*p* < 0.05). Results are expressed as mean ± SEM, * *p* < 0.05, ** *p* < 0.01.

The TMAO intervention increased MDA levels in the TMAO group in both models. Thus, in the first experiment, the intervention (F(1, 25) = 15.91, *p* < 0.0005) (Figure 5A) led to, in the TMAO + MPTP SA (*p* < 0.01) group, a significant increase in heart MDA vs. control, while, in the TMAO + MPTP SA group, it also led to an increase vs. the MPTP SA group (*p* < 0.05). In the acute model experiment, the intervention (F(1, 27) = 6.208, *p* < 0.0192) accounted for the increases in MDA (Figure 5B). In the TMAO + MPTP acute group, significantly increased MDA levels vs. control (*p* < 0.05) were observed.

The intestinal MDA levels increased in a similar way after TMAO administration. In the semi-acute model, the intervention (F(1, 25) = 17.93, *p* = 0.0003) (Figure 5C) showed similar results in the TMAO group vs. control (Figure 5C, *p* < 0.05), in the TMAO + MPTP group (Figure 5C) vs. control (*p* < 0.05), and vs. the MPTP SA group (*p* < 0.01). In the acute model experiment, the intervention accounted for the variation (F(1, 27) = 24.11, *p* < 0.0001) (Figure 5D); in the MPTP acute and TMAO + MPTP acute groups, the intervention led to a significant increase in MDA production compared to control (*p* < 0.01) and the MPTP acute group (*p* < 0.05).

### 2.2. Cytokines

#### 2.2.1. Liver

**Figure 6:**
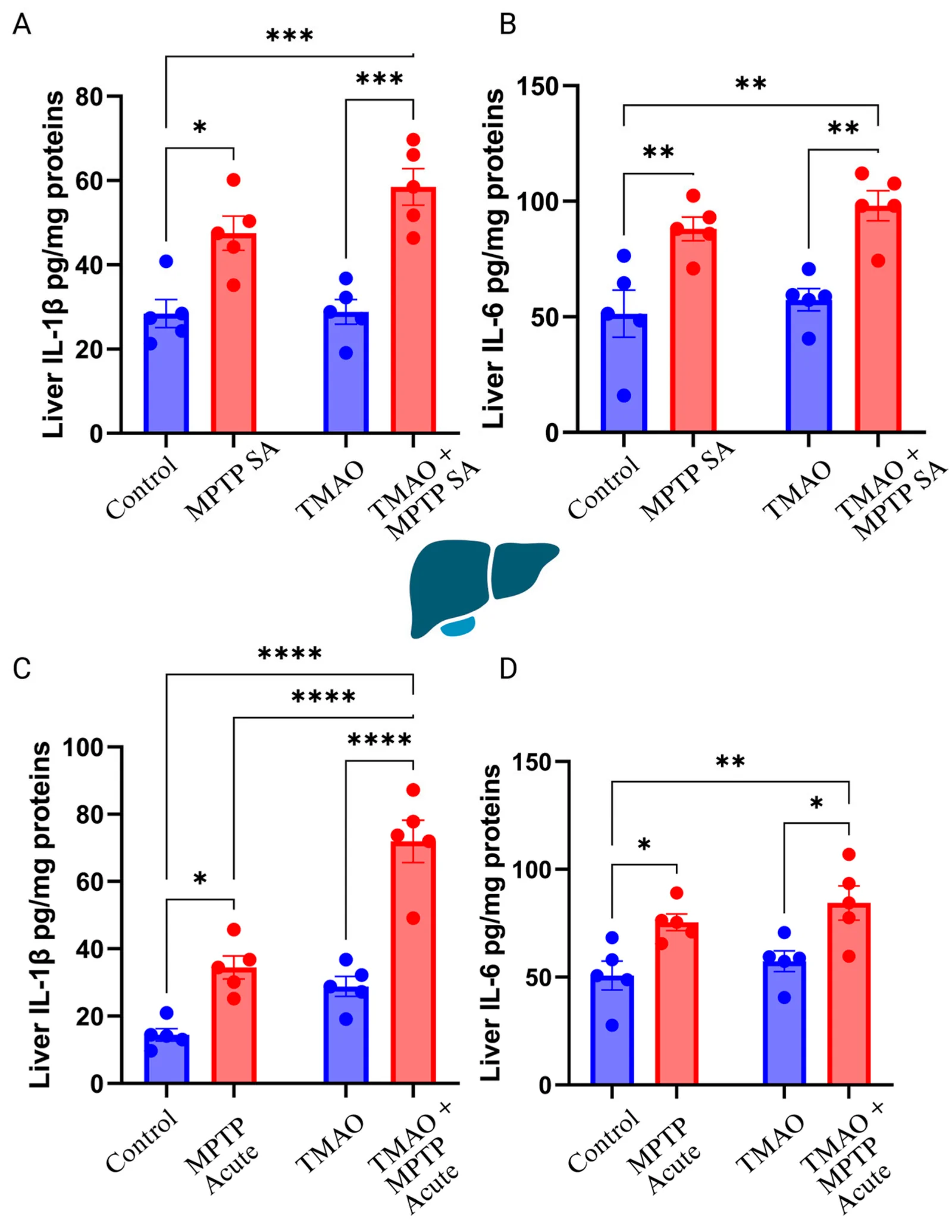
Liver cytokines. Results for the semi-acute model (**A**,**B**) (control *n* = 5; MPTP SA *n* = 5; TMAO *n* = 5; TMAO + MPTP SA *n* = 5,) and the acute model (**C**,**D**) (control *n* = 5; MPTP acute *n* = 5; TMAO *n* = 5; TMAO + MPTP acute *n* = 5) following two-way ANOVA and Tukey’s honestly significant difference (HSD) post hoc test. IL-1β is increased by TMAO + MPTP SA vs. control (*p* < 0.001) and vs. TMAO (*p* < 0.001) and by MPTP SA vs. control (*p* < 0.05) (**A**). IL-6 is also increased by TMAO + MPTP SA vs. control (*p* < 0.01) and vs. TMAO (*p* < 0.01) and by MPTP SA vs. control (*p* < 0.01) (**B**). For the acute model, IL-1β is increased in the TMAO + MPTP acute group vs. control (*p* < 0.0001), vs. TMAO (*p* < 0.0001), and vs. MPTP acute (*p* < 0.0001) and by MPTP acute vs. (**C**). IL-6 is increased by TMAO + MPTP acute vs. control (*p* < 0.01) and vs. TMAO (*p* < 0.05) (**D**). Results are expressed as mean ± SEM, * *p* < 0.05, ** *p* < 0.01, *** *p* < 0.001, **** *p* < 0.0001.

Hepatic IL-1β levels were increased by the disease model in the semi-acute model (F(1, 16) = 42.99, *p* < 0.0001) and by the intervention (F(1, 16) = 42.48, *p* < 0.0001), the acute disease model (F(1, 16) = 62.88, *p* < 0.0001) and their interaction in the disease model (F(1, 16) = 8.448, *p* = 0.0103). In the semi-acute (Figure 6A) model, TMAO + MPTP SA led to increases in IL-1β (*p* < 0.01) vs. control and vs. TMAO. For the acute model (Figure 6C), TMAO + MPTP acute led to increases for IL-1β vs. all other groups (*p* < 0.0001), while MPTP also increased it vs. control (*p* < 0.05). For the semi-acute disease model, it led to increases in IL-6 (F(1, 16) = 30.67, *p* < 0.0001) (Figure 6B). TMAO + MPTP SA increased IL-6 secretion in the liver compared to control and TMAO (*p* < 0.01), while MPTP SA increased it vs. control (*p* < 0.01). The acute model led to increases in IL-6 (F(1, 16) = 18.38, *p* = 0.0006) (Figure 6D), TMAO + MPTP acute increased liver IL-6 levels compared to control (*p* < 0.01) and vs. TMAO (*p* < 0.05), while MPTP acute increased it vs. control (*p* < 0.05).

#### 2.2.2. Kidney

**Figure 7:**
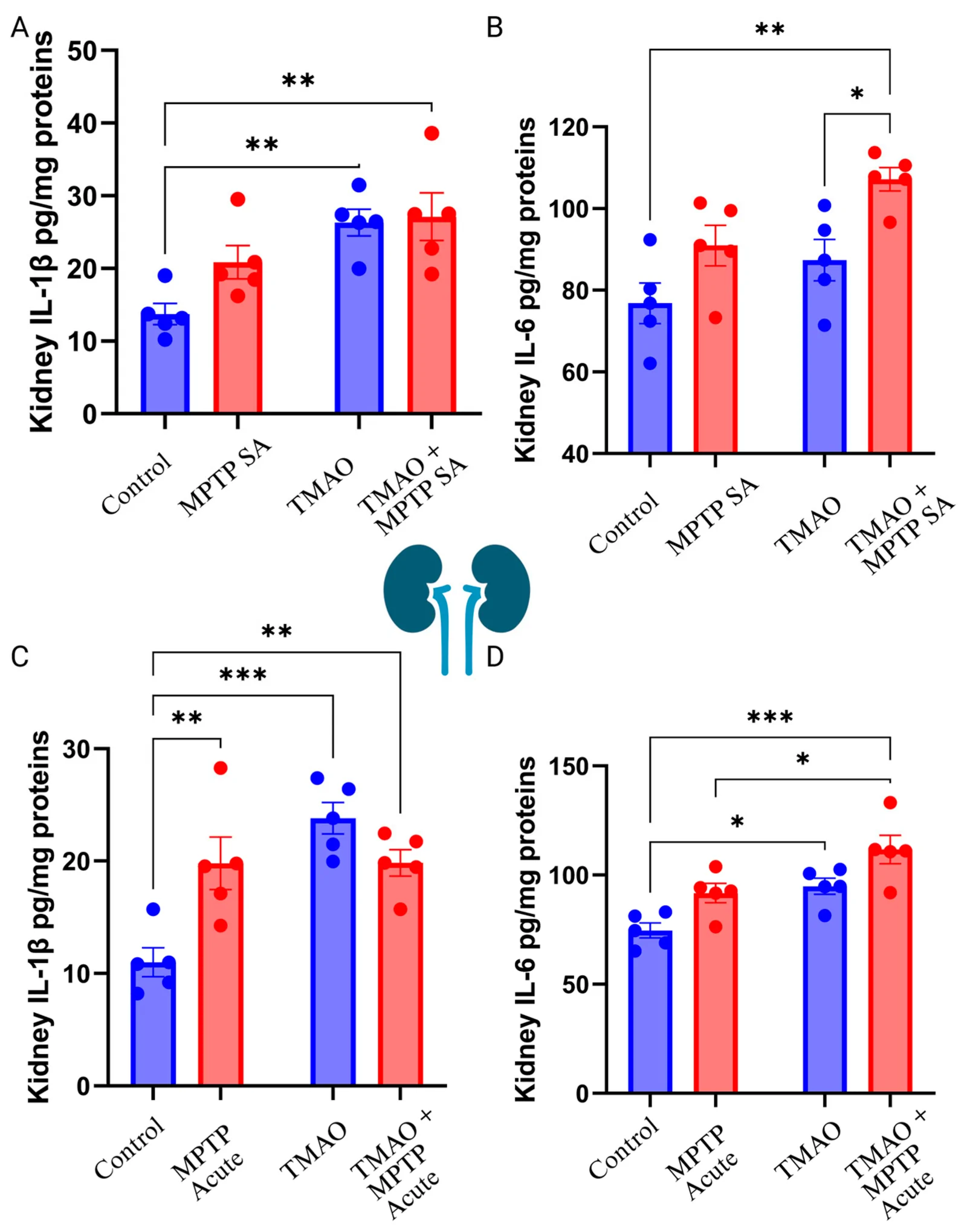
Kidney cytokines. Results for the semi-acute MPTP model (**A**,**B**) and the acute MPTP model (**C**,**D**) following two-way ANOVA and Tukey’s honestly significant difference (HSD) post hoc test (control *n* = 5; MPTP SA *n* = 5; TMAO *n* = 5; TMAO + MPTP SA *n* = 5, MPTP acute *n* = 5, TMAO + MPTP acute *n* = 5). IL-1β is increased by TMAO + MPTP acute vs. control (*p* < 0.01). TMAO led to increased levels vs. control (*p* < 0.01) (**A**). IL-6 is also increased by TMAO + MPTP acute vs. control (*p* < 0.01) and vs. TMAO (*p* < 0.05) (**B**). For the acute model, IL-1β is increased in the TMAO + MPTP acute group vs. control (*p* < 0.01). TMAO (*p* < 0.001) and MPTP acute (*p* < 0.01) also led to an increase vs. control (**C**). IL-6 is increased by TMAO + MPTP acute vs. control (*p* < 0.01) and vs. MPTP acute (*p* < 0.05) (**D**). Results are expressed as mean ± SEM, * *p* < 0.05, ** *p* < 0.01, *** *p* < 0.001.

As opposed to the liver cytokine levels, the IL-1β levels were increased in the semi-acute model by the TMAO intervention (F(1, 16) = 16.56, *p* = 0.0009), while in the acute model, it was the intervention (F(1, 16) = 15.72, *p* = 0.0011) and the interaction (F(1, 16) = 15.51, *p* = 0.0012) that increased the levels. For the semi-acute model, kidney IL-1β levels (Figure 7A) increased compared to the control, both in the TMAO group (*p* < 0.01) as well as in the TMAO + MPTP SA group (*p* < 0.01). For the acute model (Figure 7B), this increase was noticed in all groups compared to the control: TMAO (*p* < 0.001), MPTP acute (*p* < 0.01), TMAO + MPTP acute (*p* < 0.01). In the case of IL-6, the acute model also played a role (F(1, 16) = 13.17, *p* = 0.0023), along with the intervention (F(1, 16) = 18.38, *p* = 0.0006), while no interaction was found for the two. The same results were obtained for the semi-acute model, with the intervention (F(1, 16) = 8.639, *p* = 0.0096) and the disease model (F(1, 16) = 13.89, *p* = 0.0018) accounting for variation. The post hoc group comparison shows that IL-6 was increased by TMAO + MPTP SA vs. control (*p* < 0.01) and vs. TMAO (*p* < 0.05) (Figure 7C) and TMAO + MPTP acute vs. control (*p* < 0.001) and vs. MPTP acute (*p* < 0.05) (Figure 7D).

### 2.3. Histopathological Analysis

**Figure 8:**
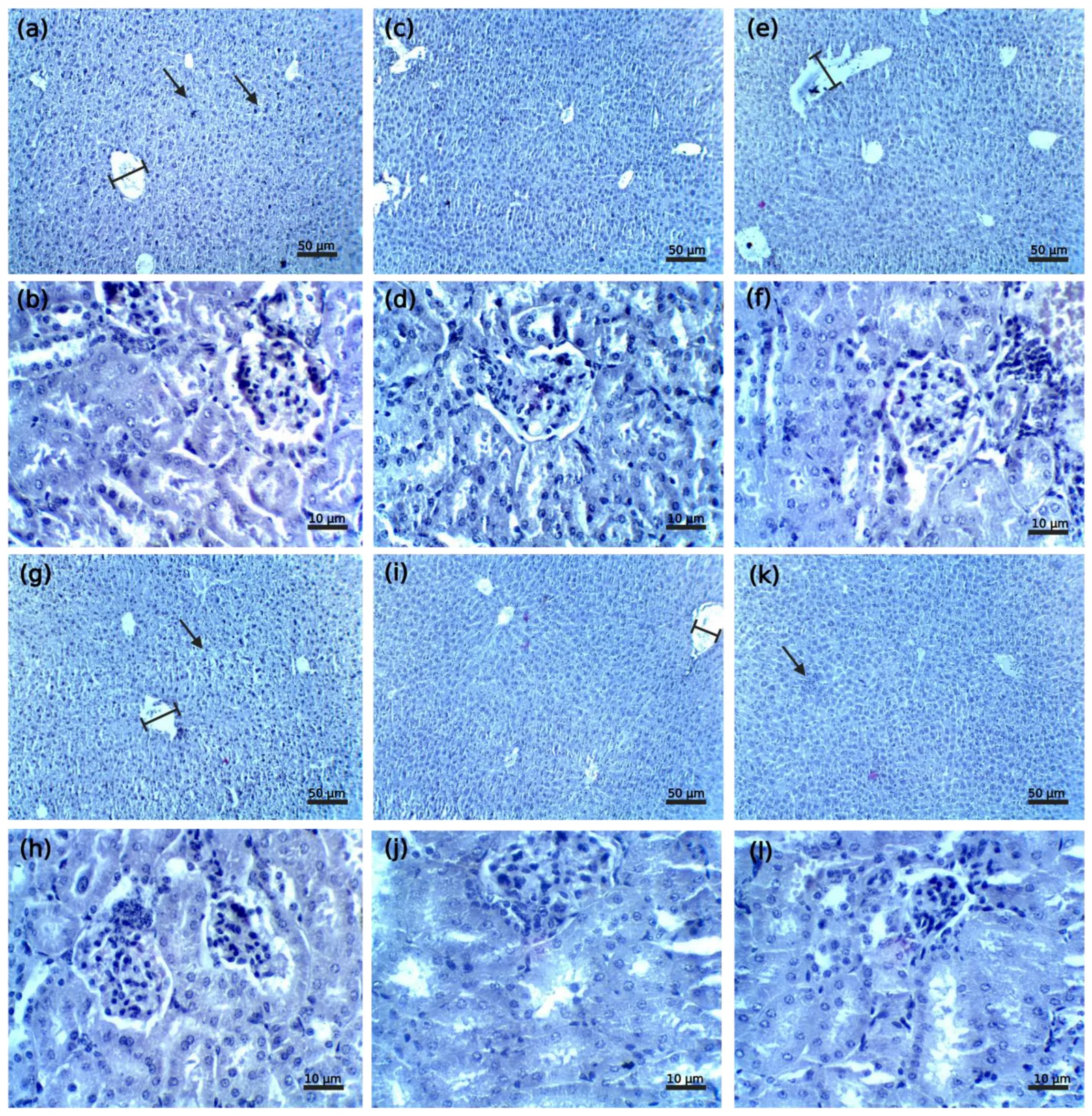
Histological analysis. Representative histological fields of the liver and kidney from the control and experimental groups. The groups are arranged as follows: control group, liver (**a**) and kidney (**b**); TMAO group, liver (**c**) and kidney (**d**); MPTP SA group, liver (**e**) and kidney (**f**); TMAO + MPTP SA group, liver (**g**) and kidney (**h**); MPTP acute group, liver (**i**) and kidney (**j**); acute TMAO + MPTP group, liver (**k**) and kidney (**l**). H&E staining: 10× objective magnification for liver sections and 40× magnification for kidney sections. Arrow shows dystrophic changes, while the segment shows the vascular diameter.

Histopathological analysis did not reveal any notable microscopic alterations in either the liver or the kidney despite the biochemical data indicating a series of changes associated with oxidative stress. Nevertheless, at the hepatic level, exposure to the TMAO + MPTP semi-acute combination (Figure 8g) resulted in a mild dystrophic pattern that appeared reversible and may be of particular relevance for the translational implications of these findings in a clinical context. The kidney appeared more vulnerable than the liver, with the most pronounced alterations occurring during the study’s subacute phase. Thus, exposure to TMAO (Figure 8d) resulted in mild tubular epithelial desquamation, whereas semi-acute exposure to MPTP (Figure 8f) was associated with focal inflammatory changes in the juxtaglomerular region, while the renal tubules otherwise retained a largely normal histological appearance. Under acute MPTP exposure, the tubular cellular mass exhibited a more pronounced eosinophilic pattern, which may represent a post-lesional response preceding potential necroptotic changes. Such alterations are consistent with the recognized susceptibility of the rodent kidney to injury, as renal tissue is particularly sensitive to a range of metabolic and toxic insults.

**Figure 9:**
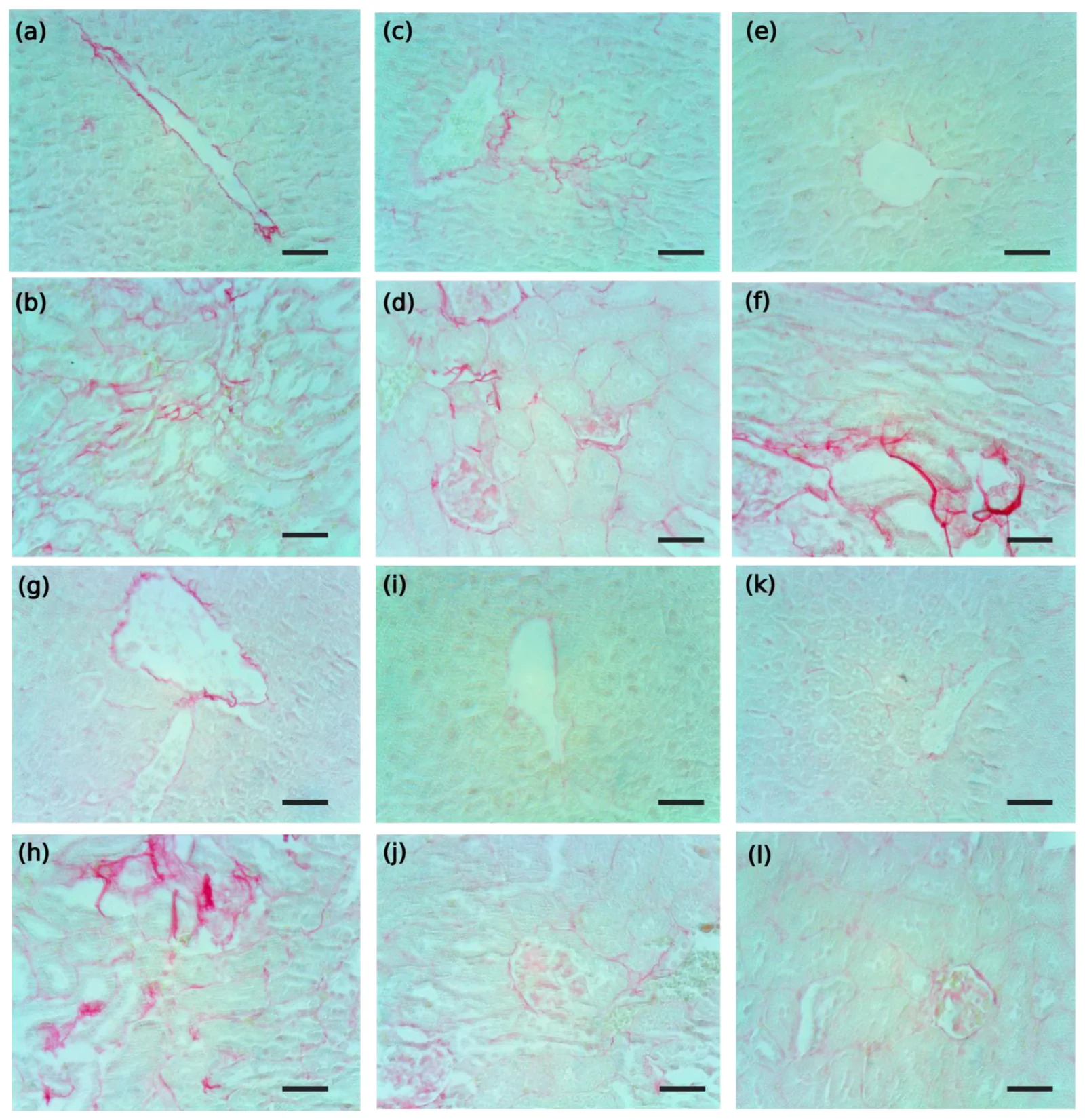
Representative Sirius Red-stained sections of the liver and kidney from the control and experimental groups. The groups are arranged as follows: control group, liver (**a**) and kidney (**b**); TMAO group, liver (**c**) and kidney (**d**); MPTP SA group, liver (**e**) and kidney (**f**); TMAO + MPTP SA group, liver (**g**) and kidney (**h**); MPTP acute group, liver (**i**) and kidney (**j**); acute TMAO + MPTP group, liver (**k**) and kidney (**l**). In the kidney, increased periglomerular collagen deposition and mild-to-moderate tubular alterations were observed, particularly following subacute MPTP exposure (**f**). In the liver, collagen deposition was restricted to areas considered within the normal histological range, with no evident increase in vascular or perivascular collagen deposition suggestive of hepatic fibrosis. Scale bar = 50 μm; 25× magnification.

The integrated analysis of H&E (Figure 8) and Sirius Red staining (Figure 9) allowing for the detailed assessment of hepatic collagen deposition, was summarized using the Knodell scoring system (Table 1), and kidney features were summarized in Table 2, based on the EGTI score. Based on the histological findings, the liver exhibited significant alterations following acute MPTP exposure. In contrast, TMAO exposure, subacute MPTP exposure, and the combined TMAO + MPTP treatments, both subacute and acute, did not induce notable hepatic lesions according to the Knodell scoring system, which evaluates periportal and bridging necrosis, intralobular degeneration and focal necrosis, portal inflammation, and fibrosis. At the kidney level, subacute exposure to MPTP induced histopathological changes that reached a score of 5 on the EGTI scale (maximum score = 14). These alterations were characterized by mild endothelial swelling, thickening of the tubular basement membrane affecting more than 25% of tubular cells, and the presence of collagen deposits, as evidenced by Sirius Red staining. In the liver, collagen deposition was confined to areas consistent with normal histological architecture, with no evident increase in vascular or perivascular collagen deposition indicative of hepatic fibrosis.

## 3. Discussion

### 3.1. Main Findings

Our results can be categorized into three types: the ones that were caused by the MPTP disease model, others that were caused by the atherosclerotic TMAO dose, and results that were attributed to an interaction of the two.

#### 3.1.1. Effects of the Disease Model

Both models of MPTP lead to a depletion in SOD activity in the liver, showing indirectly the oxidative stress generated in the hepatic tissue. This is followed by a diminution in GSH levels and an increase in GSSG and MDA production, but only in the TMAO + MPTP SA and TMAO + MPTP acute groups. These redox imbalances were only partially reflected in the cytokine levels. In the liver, semi-acute IL-1β was significantly increased by both the disease model and the intervention as independent main effects, with TMAO + MPTP SA significantly elevated versus both control and TMAO alone (*p* < 0.01); no significant interaction was identified for this comparison.

In the kidney, the semi-acute model showed a complex pattern: GSH and GSSG levels individually were not significantly altered by either the disease model or the intervention. However, the GSH/GSSG ratio was significantly reduced, driven by combined contributions of the disease model and the intervention. The semi-acute disease model did significantly contribute to increased MDA, alongside the intervention, with the TMAO + MPTP SA group showing the most pronounced elevation versus the control and MPTP alone. In the acute model, by contrast, TMAO intervention was the sole significant contributor to both SOD depletion and MDA elevation, with no significant contribution from the disease model itself. Taken together, these findings indicate that MPTP’s contribution to kidney redox imbalance is model-specific: in the semi-acute regimen, MPTP meaningfully contributes to both MDA elevation and glutathione ratio disruption, whereas in the acute regimen, TMAO alone accounts for the observed oxidative changes, with MPTP’s effect confined to its interaction with GSSG and the GSH/GSSG ratio.

#### 3.1.2. Effects of the TMAO Intervention

In addition to the already-mentioned effects that atherosclerotic TMAO doses induce when combined with MPTP, they also produce effects on their own. Significant effects were triggered by TMAO on the liver, kidney, heart, and intestine. Kidney SOD activity was depleted by TMAO, and IL-1β was increased in both experiments. Kidney MDA, heart MDA, and intestine MDA increased in all TMAO-receiving groups, with MPTP showing no effect on lipid peroxidation at these levels.

#### 3.1.3. Effects of the Interaction of the Disease Model and the Intervention

In the acute model, the IL-1β level was driven by a significant main effect of the disease model together with a significant disease model × intervention interaction, consistent with a true synergistic effect: TMAO + MPTP acute was significantly elevated versus all other groups, while MPTP alone was also significantly increased versus the control. For IL-6, only a significant main effect of the disease model was identified in both the semi-acute models, with no interaction reported; post hoc testing showed increases in both the MPTP-alone and combined TMAO + MPTP groups relative to the control in each model, indicating an additive rather than statistically confirmed synergistic pattern for this cytokine. Limited differences were observed between the two MPTP models. Thus, the acute model induces more extensive liver and kidney redox imbalances without the combination with TMAO, while the same level of redox imbalance was obtained in the semi-acute model only by the combination of TMAO and MPTP.

#### 3.1.4. Histological Results

Interestingly, the histopathological findings only partially mirrored the biochemical redox and inflammatory disturbances observed in the liver and kidney. Despite the absence of overt microscopic damage in most groups, the TMAO + MPTP SA combination produced a mild, seemingly reversible dystrophic pattern in the liver, potentially reflecting an early morphological correlate of the combined oxidative burden described above and one that may carry translational relevance given the reversible nature of comparable lesions in clinical hepatotoxicity. The kidney displayed greater histological vulnerability than the liver, consistent with its heightened sensitivity to biochemical redox imbalance observed in our models. The tubular epithelial desquamation associated with TMAO alone, together with the focal juxtaglomerular inflammatory changes seen under MPTP SA, suggests that distinct mechanistic pathways—metabolic/toxic versus inflammatory—may independently converge in renal injury, even in the relative absence of pronounced biochemical alterations at this stage. The more pronounced tubular eosinophilic changes observed under MPTP acute exposure, potentially representing an early post-lesional or pre-necroptotic state, may help explain the more extensive redox disturbances previously noted in our model.

The semi-quantitative histopathological scoring further refined our understanding of organ-specific vulnerability to MPTP and TMAO exposure. Notably, the liver showed significant Knodell-scored alterations exclusively under acute MPTP exposure, whereas neither TMAO alone nor the combined TMAO + MPTP regimens—acute or subacute—produced notable hepatic lesions by this scoring system. Acute MPTP exposure produces a more immediate architectural insult, while the observed inflammatory TMAO + MPTP interraction does not lead to scorable histological damage.

It should be noted that the histopathological findings presented here are primarily descriptive in nature. While semi-quantitative scoring systems (Knodell for the liver, EGTI for the kidney) were applied to provide a structured and reproducible framework for comparison, these scores are intended to characterize the pattern and severity of observed changes rather than to establish definitive causal or mechanistic links between TMAO/MPTP exposure and organ injury. As such, these findings should be interpreted as hypothesis-generating observations that complement, rather than independently confirm, the biochemical and inflammatory results reported above.

Taken together, these histopathological observations suggest that peripheral organ vulnerability to MPTP and TMAO exposure may not be uniform, with the kidney potentially representing a comparatively more susceptible site for structural changes.

### 3.2. Relevance in the Context of Previous Research

Our study focused on investigating MPTP’s effect on peripheral organs combined with the gut-derived metabolite TMAO, in the context of increasing evidence of the role of the kidneys [24,34] and liver [35,36] in the progression of PD.

The peripheral and central immune systems are not isolated—they interact dynamically throughout PD progression. Environmental triggers stimulate peripheral immune cells, including T cells and macrophages, to induce secretion of proinflammatory cytokines such as TNF-α, IL-1β, and IL-6, sustaining chronic systemic inflammation [30]. The cytokines can compromise blood–brain barrier integrity by downregulating tight junction proteins such as claudin-5 and occludin, ultimately contributing to neuroinflammation in the CNS and potentially driving the progression of neurodegenerative diseases such as PD [30]. This could be relevant for our findings, as IL-1β and IL-6 were elevated in the kidney and liver, especially in the context of receiving TMAO and MPTP. While our findings are consistent with a model in which peripheral oxidative stress and inflammation—particularly the elevated IL-1β and IL-6 observed in the liver and kidney under combined TMAO + MPTP exposure—may contribute to or exacerbate central neuroinflammatory and neurodegenerative processes, this remains a hypothesis that our current data cannot directly test. Future studies incorporating these elements are needed to determine whether a causal link can be established.

Peripheral oxidative stress does not propagate directly to the brain in the form of ROS. But there is indirect propagation present, through activation of pathways, especially NF- κB in peripheral and organ-resident immune cells, resulting in increases of proinflammatory cytokines like the aforementioned IL-1β and IL-6 [37].

Beyond cytokines, oxidative stress can itself disrupt the BBB [38]. So, while ROS themselves cannot travel to the brain, they can weaken the BBB locally in cerebrovascular endothelial cells, which are exposed to circulating blood and therefore to peripheral oxidative signals [38].

A population-based study found that TMAO increases the risk of dementia, and precursors of TMAO were associated with poor cognition [18]. Since renal clearance is important in the case of this osmolyte, the study assessed how kidney function might affect TMAO concentrations and if this would be associated with dementia [18]. They have found that impaired renal clearance leads to increased risk of dementia with increased TMAO, while for those who have preserved renal function, only the precursor levels might be related to dementia [18].

TMAO activates nuclear factor kappa-B (NF-κB) nuclear translocation, resulting in the upregulation of NLRP3 inflammasome and other proinflammatory genes through ROS stimulation [39,40]. The NLRP3 inflammasome leads to macrophage activation and can also be directly activated by TMAO stimulating IL-1β secretion [41]. Also, TMAO produces ROS in macrophages, thus creating a loop that amplifies oxidative stress and inflammation [40]. TMAO leads to kidney inflammation, oxidative stress, and apoptosis [42,43]. Also, through its well-known effect on the circulatory endothelium, TMAO might also lead to decreased nitric oxide availability and vasoconstriction [44]. Moreover, TMAO alters renal tubular exchange [45]. These effects help explain the previously observed association between elevated TMAO levels and acute kidney injury and chronic kidney disease [46].

Our findings partially fit in the context of previously published research, showing that TMAO increased the redox imbalance in the kidneys and amplified inflammation and IL-1β secretion. The MPTP SA model was not associated with redox imbalances while the acute MPTP administration induced a depletion in GSH, an increase in GSSG, and a reduction in the GSH/GSSG ratio.

TMAO was also previously associated with chronic liver disease states, such as cirrhosis, non-alcoholic fatty liver disease, and subsequent hepatic encephalopathy [36]. This happens through intestinal dysbiosis, especially decreased Bacteroides and increased Proteobacteria, which results in reduced short-chain fatty acids (SCFAs) and increased TMAO. These are important regulators that connect the liver disease and PD [47]. Increases in the TMA-producing bacteria, as well as higher intake of choline, L-carnitine, and betaine, lead to increased TMA production [48]. Coupled with liver inflammation, this increased in the activity of enzymes that convert TMA to TMAO, with a gradual decrease in the function of the liver that reduces TMAO clearance, leading to an increase in TMAO in serum [49]. Epidemiologically, PD patients with liver dysfunction show higher plasma ammonia levels [50]. Interestingly, MPTP models are associated with increased ammonia concentrations due to reduced activity of glutamine synthetase in astrocytes [51]. Given this association and our findings regarding the impact of MPTP on liver oxidative stress, further research is necessary to assess whether liver function is affected before and after MPTP modeling. This interaction might also help partially explain inter-individual variability in MPTP models.

In our study, 40 mg/kg TMAO, administered once daily, was not enough to increase liver oxidative stress. This could be either due to the lower, atherosclerotic dose or to the limited exposure time. On the other hand, both the semi-acute and the acute MPTP models did deplete liver SOD activity, but this did not translate to glutathione imbalances, lipid peroxidation, or an increase in inflammatory markers. Only the combined groups in acute or subacute models lead to alterations in the glutathione and lipid peroxidation, with MPTP depleting SOD and TMAO causing subsequent imbalances. This is further sustained by the inflammatory markers, as IL-1β and IL-6 levels were only increased in the combined TMAO and MPTP groups.

Previous studies have shown that TMAO was associated with higher mortality in heart failure patients [52,53]. The exact relationship is yet unclear, as it might indicate comorbidities such as kidney dysfunction rather than direct harm [54].

In the intestine, TMAO triggers inflammasome activation and ROS production in a dose-and time-dependent manner [42]. This is highly relevant, since oxidative stress and inflammation at this level will affect the gut–blood barrier and result in increased absorption of proinflammatory molecules [55]. In our findings, we have found increased lipid peroxidation produced by TMAO. Further studies should be performed to identify the inflammatory response to an atherosclerotic dose of TMAO.

Our findings demonstrated that TMAO consistently drives lipid peroxidation across peripheral organs (kidney, heart, intestine), regardless of the disease model, with the liver being affected only in the context of combination with MPTP.

A systematic review and meta-analysis of 80 studies encompassing over 7000 PD patients found that blood MDA concentrations were significantly increased in patients with PD compared to healthy controls, making it one of the most robustly replicated peripheral oxidative stress findings in PD [56].

The same study found that plasma antioxidant status such as SOD activity is impaired in PD patients, with plasma SOD levels in PD patients remarkably lower than in healthy controls, indicating that lower plasma levels of SOD may contribute to the neuropathogenesis of PD and may be correlated with disease severity [57].

The systematic meta-analysis showed that oxidized glutathione was significantly higher in red blood cells of PD patients, although there were no changes in total glutathione and reduced glutathione compared to controls [56]. This is a pattern similar to what happened in our kidney tissue in the semi-acute model for the groups that received TMAO and MPTP.

The natural progression of oxidative stress and inflammation in PD is characterized by rising MDA, altered glutathione (particularly lowered GSH/GSSG ratio), variable but generally declining SOD in plasma, and consistently elevated IL-1β and IL-6 that scale with disease severity [56]. Our combined TMAO + MPTP groups recapitulate this profile in peripheral organs, showing elevated MDA, disrupted glutathione homeostasis, depleted SOD, and elevated IL-1β and IL-6 in the liver and kidney.

A missing piece that is relevant for our experimental setup is the environmental exposure to toxins as a possible cause of PD [57]. If a toxin can create simultaneous dysbiosis with bacteria that produce TMA, while also inducing mithocondrial dysfunction, it can be argued, based on our results that show significant oxidative and inflammatory changes, that changes might be observed in the kidney and the liver, with possible downstream effects. Further studies should be performed to map this theoretical environmental exposure and to determine if peripheral organ effects would also result in effects on dopaminergic neurons.

Central nervous system samples from the same animals were processed and evaluated independently, and the corresponding neuropathological and biochemical findings have been published elsewhere [58]. Our peripheral findings can now be interpreted in light of the central nervous system data reported in our companion study using the same experimental cohorts. In that work [58], motor behavioral testing confirmed MPTP-induced impairment across rotarod, wire hang, and pole test parameters, with a combined effect between TMAO and MPTP observed specifically in pole-turning time. Critically, a significant reduction in midbrain tyrosine hydroxylase (TH) expression was observed exclusively in the TMAO + MPTP group, mirroring the pattern we describe here in the liver and kidney, where oxidative and inflammatory alterations were also most pronounced, or exclusively present, in the combined TMAO + MPTP groups. Notably, neuroinflammatory profiling revealed selective elevation of IL-6 in the TMAO + MPTP group, without accompanying microglial activation, whereas NF-κB p65 was paradoxically increased only in the TMAO and MPTP groups alone, a pattern the authors hypothesized may reflect a shift toward necrotic rather than apoptotic cell death in the combined exposure group [58].

### 3.3. Limitations and Future Research

When it comes to limitations, one of the biggest is the lack of data for cytokines for heart and intestinal tissue. This is doubled by the other big flaw of our study: the lack of multiple timepoints that could offer a possibility for temporal relations to be observed. Further studies should be performed to identify whether an inflammatory response is also present in the heart and intestine and whether it has a time dependence. The previously described effect of MPTP in heart tissue was not replicated in our study. This might be due to a lack of markers or a temporal dynamic that was not observable in our design. Another flaw is the lack of functional outcome data to assess the liver, kidney and cardiac function. This limits the interpretation of our results only to observed oxidative stress and inflammation, as we were not able to identify if downstream organ impairments are associated. Furthermore, our single-timepoint analysis at sacrifice precludes any assessment of the temporal dynamics of these redox and inflammatory changes, limiting our ability to distinguish transient adaptive responses from sustained pathological alterations and to establish whether the observed peripheral effects precede, coincide with, or follow potential central changes.

Future work should expand upon our findings and identify the mechanistic pathways, such as NF-κB and NLRP3 inflammasome, that are upstream of our observed oxidative stress and inflammation, as well as gut microbiome profiling and functional outcomes, to observe the upstream impact on TMAO-producing bacteria. Future studies incorporating multi-organ cytokine panels alongside longitudinal sampling across the disease induction period would allow for a more complete mechanistic and temporal understanding of the interaction between TMAO and MPTP and could help clarify the sequence of peripheral events relative to central neurodegeneration.

## 4. Materials and Methods

### 4.1. Animal Grouping, Disease Induction, Intervention and Timeline

A total of 96 CD-1 male mice, aged 6–8 weeks (32.98 ± 0.87 g), were obtained from the Biobase of the “Iuliu Hațieganu” University of Medicine and Pharmacy, Cluj-Napoca; half were obtained for one experimental setting and half for the other. Animals were randomly allocated into 8 groups and maintained under controlled environmental conditions (temperature: 24 ± 2 °C; humidity: 55 ± 10%) on a 12 h light/dark cycle, with unrestricted access to water and standard rodent chow. Following arrival, a 7-day acclimatization period was observed before experimental procedures commenced. The overall experimental timeline, which is common for both experiments, is illustrated in Figure 8.

**Figure 10:**
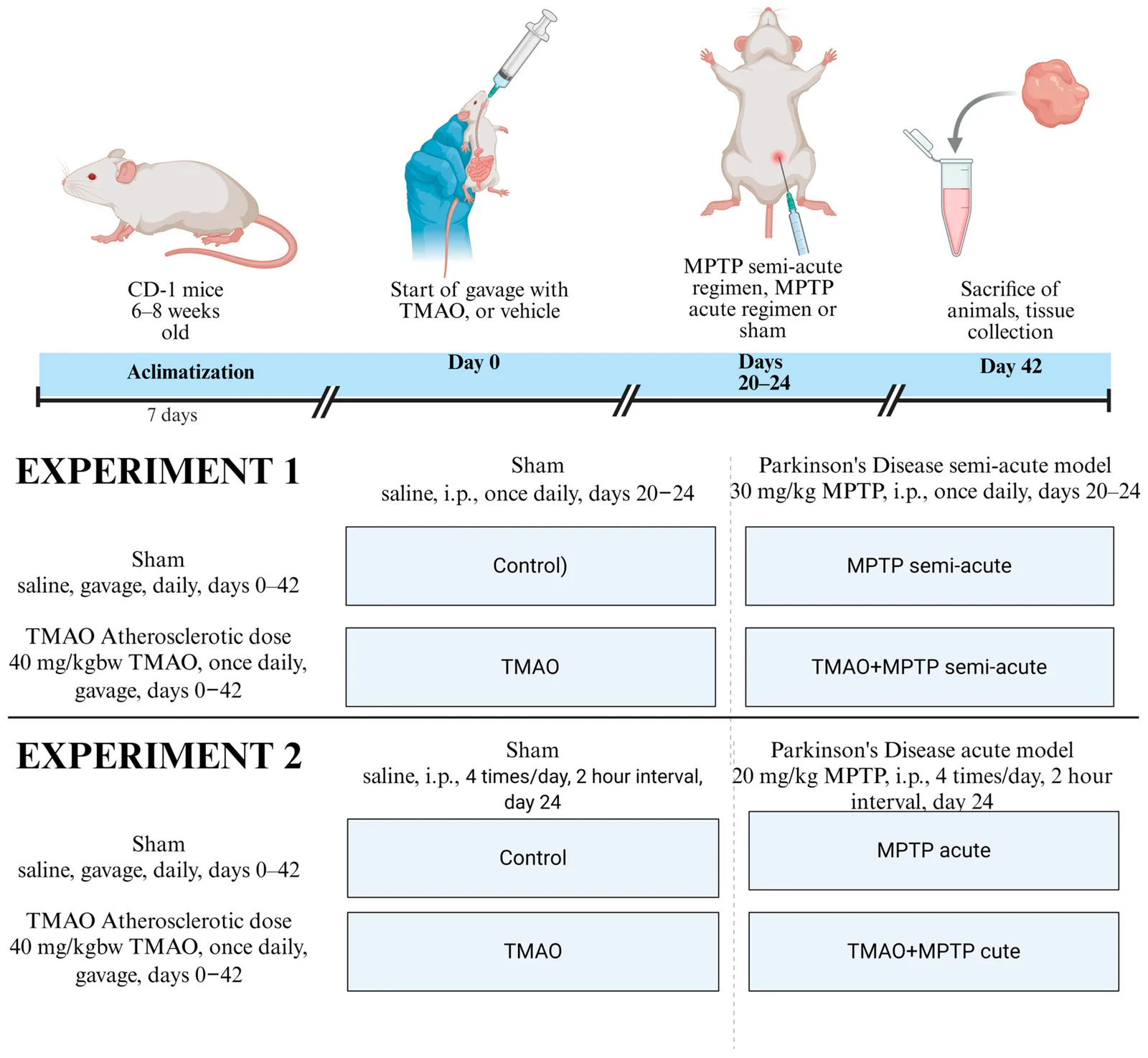
Timeline of the experiments and experimental groups. Experiment 1—control, TMAO, MPTP semi-acute, TMAO + MPTP semi-acute; Experiment 2—control, TMAO, MPTP acute, TMAO + MPTP acute.

The animals were randomized in the 2 experiments, totaling 8 groups (Figure 10), as can be seen in Figure 8. The first experiment consisted of control group (12 animals) which received saline both for the disease induction (once daily, i.p, days 20–24), as well as saline for intervention (saline, gavage, once daily, days 0–42); a TMAO group (12 animals) which received saline for the disease model and TMAO atherosclerotic dose (40 mg/kg TMAO, once daily, gavage, days 0–42); an MPTP group (12 animals) which received the semi-acute regimen of MPTP and saline by gavage, daily, days 0–42; and an M + T+ group (12 animals) that received both 30 mg/kg MPTP, i.p., once daily, days 20–24, and 40 mg/kg TMAO, once daily, gavage, days 0–42.

The second experiment consisted of the following groups: control (12 animals) which received saline, i.p., once daily, four times at 2-h intervals, day 24, as well as saline for the intervention (gavage, once daily, days 0–40)**;** TMAO group (12 animals) which received saline and TMAO atherosclerotic dose (40 mg/kg bw once daily, gavage, days 0–42); MPTP group (12 animals) which received the acute regimen of MPTP (20 mg/kg bw MPTP, i.p, four times at 2-h intervals, day 24) and saline, by gavage, daily, days 0–42**;** MPTP + TMAO group (12 animals) which received the acute regiment of MPTP (20 mg/kg bw MPTP, i.p, four times at 2-h intervals, day 24) and TMAO (40 mg/kg bw, once daily, gavage, days 0–42).

Regarding attrition, in the first experiment we report the death of three animals, one in each of the following groups: TMAO, MPTP SA, TMAO + MPTP SA. In the second experiment, one animal died in the course of the experiment, in the TMAO group. This resulted in the following endpoint sizes for each group: first experiment—control (12), TMAO (11), MPTP SA (11), TMAO + MPTP SA (11); second experiment—control (12), TMAO (11), MPTP acute (12), TMAO + MPTP acute (12). Out of this number of animals, 4 were allocated for histological analysis for each group, with the remaining 7–8 animals per group being allocated for oxidative analyses; 5 out of each group were randomly selected for cytokine testing.

The TMAO dose was chosen in accordance with previous studies that have established high-risk TMAO levels as over 5–6.18 µmol/L or 375–465 ng/mL [58]. We have proven in previous studies that this level can be attained by the administration of 40 mg/kg bw TMAO [59]. Previous studies have used the same dose and have reported incipient aortic atherosclerosis [60,61].

At the end of the experimental period (day 42), animals were humanely sacrificed by cervical dislocation. The kidneys, liver, heart and whole intestine were immediately dissected, with 4 animals for each group being reserved for histopathological examination and the rest stored at −80 °C for future analysis. Central nervous system findings from the same cohort are not presented here, as they have been partially reported in detail in a separate publication [58], which specifically addressed dopaminergic neuronal integrity and central markers of neuroinflammation. All the work is in accordance with the EU Directive 2010/63 on the protection of animals regarding the three Rs of ethical animal research used for scientific purposes, as well as the local legislative framework; this was certified by the approval of the Ethics Committee of the University of Medicine and Pharmacy “Iuliu Hatieganu” Cluj-Napoca (DEP119/11.04.2023) and the National Sanitary Veterinary and Food Safety Authority (A.N.S.V.S.A) with the project authorization number 381 from 25 August 2023.

### 4.2. Oxidative Stress Parameters

Homogenisation of kidney, liver, heart, and intestinal tissue (*n* = 7–8/group) was performed on ice (POLYTRON PT 1200 E, Kinematica, Malters, Switzerland) in 50 mM TRIS and 10 mM EDTA buffer at pH 7.5. Resulting samples were centrifuged (1000× *g*, 10 min, 4 °C), and the supernatant was collected and stored in pre-labeled sterile Eppendorf tubes, transported on crushed ice, and immediately frozen at −80 °C until further analysis. Sample protein content was determined through the Bradford method [62].

Oxidative stress parameters were determined as follows: liver and kidney samples’ SOD activity, MDA, GSH and GSSG levels, as well as GSH/GSSG ratio; heart and intestine for quantification of MDA levels. MDA levels, as markers of polyunsaturated fatty acid peroxidation end products, were determined fluorometrically according to the protocol described by Conti et al. [63]. Following extraction with *n*-butanol, MDA content was measured spectrofluorometrically at excitation/emission wavelengths of 534/548 nm (Perkin Elmer, Shelton, CT, USA) and expressed as nmol/mL and nmol/mg protein.

GSH and GSSG concentrations were measured as previously described [64]. The GSH/GSSG ratio was also calculated as a well-established index of oxidative stress status.

### 4.3. Cytokines

Liver and kidney IL-1β (*n* = 5/group) and IL-6 (*n* = 5/group) levels were measured using mouse-specific ELISA kits as per the manufacturer’s instructions (E-EL-M3063; Elabscience, Houston, TX, USA). Absorbance readings were performed at 450 nm, and the results were expressed as pg/mg protein.

### 4.4. Histopathological Examination

For histopathological analysis, tissue samples (*n* = 4 per group) were collected immediately following euthanasia and fixed in 5% neutral-buffered formalin at room temperature to ensure adequate tissue preservation. The tissues were subsequently embedded in paraffin blocks and allowed to solidify at room temperature. Paraffin-embedded blocks were sectioned at a thickness of 5 μm before being mounted onto glass microscope slides. The sections were deparaffinized in xylene and rehydrated through a descending ethanol series to distilled water. Slides were then subjected to routine hematoxylin–eosin (H&E) staining. Histological evaluation was performed by light microscopy, assessing general tissue architecture, cellular morphology, and any pathological alterations. Collagen deposition in liver and kidney tissue sections was evaluated using Sirius Red staining. Briefly, paraffin-embedded tissue sections (5 μm thick) were deparaffinized in xylene and rehydrated through a graded ethanol series to distilled water. The sections were then stained with Sirius Red solution, allowing selective binding of the dye to collagen fibers. Following staining, the sections were rinsed to remove excess dye, dehydrated through graded ethanol, cleared in xylene, and mounted with a suitable mounting medium. Stained sections were examined by light microscopy, and collagen deposition was assessed based on the intensity and distribution of Sirius Red-positive areas, with particular attention to vascular, perivascular, and interstitial regions.

### 4.5. Statistical Analysis

Statistical analyses were conducted using GraphPad Prism (version 9.3.0, GraphPad Software, San Diego, CA, USA; https://www.graphpad.com/) on a Windows 11 operating system. Group differences were evaluated using two-way ANOVA, with disease model (MPTP) and intervention (TMAO) as independent factors. Where the ANOVA revealed a statistically significant main effect or interaction, post hoc pairwise comparisons were performed using Tukey’s honestly significant difference (HSD) test to correct for multiple comparisons; results are reported as (F(df), *p*). Unless otherwise specified, results are expressed as mean ± SEM, with a *p*-value threshold of 0.05 considered statistically significant. Raw data can be found in Supplementary Materials.

## 5. Conclusions

Our study investigated the peripheral effects of MPTP and the gut-derived metabolite TMAO across the liver, kidney, heart and intestine, with a particular focus on the growing evidence implicating the kidney and liver in PD progression.

No significant interaction was found for any oxidative stress markers in the heart, intestine, liver or kidney in either model, nor for IL-6 in either organ. Significant interactions were confined to IL-1β in the acute model, in both the liver and kidney.

In the liver, MPTP depleted SOD in both models, while TMAO contributed to several oxidative and glutathione-related outcomes, particularly in the acute model. Both MPTP and the TMAO + MPTP combination increased liver IL-1β and IL-6, with the combined groups showing the largest increases. For acute liver IL-1β, the significant interaction and the elevation in the combined group above all other groups indicate a supra-additive response. In the kidney, the contributions were model-dependent: in the semi-acute regimen, MPTP and TMAO each contributed to MDA elevation and GSH/GSSG disruption, whereas in the acute regimen, TMAO was the main driver of SOD depletion and MDA elevation. For acute kidney IL-1β, the significant interaction reflects a synergistic response. TMAO also independently increased lipid peroxidation in the heart and intestine.

Overall, TMAO and MPTP act through organ-, model- and marker-specific, predominantly additive effects, with statistically supported interactions limited to IL-1β in the acute model. These findings are limited by single-timepoint sampling, the absence of cytokine data for the heart and intestine, and the lack of functional organ outcomes. They remain compatible with the hypothesis that a gut-derived metabolite and a neurotoxic insult together increase peripheral oxidative stress and inflammation, with possible downstream central effects. If an environmental toxin simultaneously favored TMA-producing bacteria and caused mitochondrial dysfunction, similar peripheral changes could be expected. This hypothesis warrants further investigation.

## Figures and Tables

**Table 1:** Synopsis of the liver histological evaluation of the control group and experimental groups, taking into account the Knodell score and the mean vascular wall thickness (VT), as well as the type and percentage of hepatic lesions observed.

Experimental Group	VT μm	Lesion/Area %	Fibrosis	n	Knodell Score	Mean ± SD	*p*-Value
Control (a)	-	-/0	0	4	1	1 ± 0.010	-
TMAO (c)	-	-/0	0	4	1	1 ± 0.050	>0.05
MPTP subacute (e)	57 ± 4150	dystrophy/10	0	4	2	2 ± 0.018	>0.05
MPTP acute (g)	50 ± 1.75	dystrophy/30	0	4	3	3 ± 0.093	*
TMAO + MPTP subacute (i)	52 ± 2.30	-/0	0	4	0	0	>0.05
TMAO + MPTP acute (k)	-	-/0	0	4	0	0	>0.05

VTM: vascular thickness; n: number of replicates; *p*-value was calculated for the Knodell score using *t*-test, and each experimental group was compared to control; *p* < 0.05 was considered significant and noted as follows: *p* < 0.05 (*).

**Table 2:** Synopsis of the kidney histological evaluation of the control group and experimental groups, taking into account the EGTI score (endothelial, glomerular, tubular, and interstitial components).

Experimental Group	Endothelial	Glomerular	Tubular	Tubulo/Interstitial	n	EGTI Score	Mean ± SD	*p*-Value
Control (b)	0	0	0	0	4	0	0	-
TMAO (d)	0	0	1	1	4	2	2 ± 0.030	*
MPTP subacute (f)	1	0	2	2	4	5	5 ± 0.180	***
MPTP acute (h)	0	0	0	0	4	0	0	>0.05
TMAO + MPTP subacute (j)	0	0	0	0	4	0	0	>0.05
TMAO + MPTP acute (l)	0	0	0	0	4	0	0	>0.05

*p*-value was calculated for the EGTI score using *t*-test, and each experimental group was compared to control; *p* < 0.05 was considered significant and noted as follows: *p* < 0.05 (*), *p* < 0.001 (***).

## Data Availability

Available upon reasonable request.

## Supplementary Materials

The following supporting information can be downloaded at: https://www.mdpi.com/article/10.3390/ijms27209377/s1.

## Abbreviations

The following abbreviations are used in this manuscript:

PD	Parkinson’s disease
MPTP	1-methyl-4-phenyl-1,2,3,6-tetrahydropyridine
TMAO	trimethylamine N-oxide
SOD	superoxide dismutase
IL-1β	interleukin-1 beta
IL-6	interleukin-6
MDA	malondialdehyde
ROS	reactive oxygen species
TMA	trimethylamine
FMO	flavin monooxygenase
GSH	reduced glutathione
GSSG	oxidized glutathione
MPTP SA	1-methyl-4-phenyl-1,2,3,6-tetrahydropyridine semi-acute model
MPTP acute	1-methyl-4-phenyl-1,2,3,6-tetrahydropyridine acute model
